# Chronic Thromboembolic Pulmonary Hypertension – What Have We Learned From Large Animal Models

**DOI:** 10.3389/fcvm.2021.574360

**Published:** 2021-04-16

**Authors:** Kelly Stam, Sebastian Clauss, Yannick J. H. J. Taverne, Daphne Merkus

**Affiliations:** ^1^Department of Cardiology, Erasmus University Medical Center Rotterdam, Rotterdam, Netherlands; ^2^Department of Medicine I, University Hospital Munich, Ludwig-Maximilians University Munich, Munich, Germany; ^3^Institute of Surgical Research at the Walter-Brendel-Centre of Experimental Medicine, University Hospital, LMU Munich, Munich, Germany; ^4^DZHK (German Centre for Cardiovascular Research), Partner Site Munich, Munich Heart Alliance, Munich, Germany; ^5^Department of Cardiothoracic Surgery, Erasmus University Medical Center Rotterdam, Rotterdam, Netherlands

**Keywords:** CTEPH, pulmonary hypertension, pulmonary vasculature, vascular resistence, large animal models, swine models, arrhythmogenesis, cardiac remodeling

## Abstract

Chronic thrombo-embolic pulmonary hypertension (CTEPH) develops in a subset of patients after acute pulmonary embolism. In CTEPH, pulmonary vascular resistance, which is initially elevated due to the obstructions in the larger pulmonary arteries, is further increased by pulmonary microvascular remodeling. The increased afterload of the right ventricle (RV) leads to RV dilation and hypertrophy. This RV remodeling predisposes to arrhythmogenesis and RV failure. Yet, mechanisms involved in pulmonary microvascular remodeling, processes underlying the RV structural and functional adaptability in CTEPH as well as determinants of the susceptibility to arrhythmias such as atrial fibrillation in the context of CTEPH remain incompletely understood. Several large animal models with critical clinical features of human CTEPH and subsequent RV remodeling have relatively recently been developed in swine, sheep, and dogs. In this review we will discuss the current knowledge on the processes underlying development and progression of CTEPH, and on how animal models can help enlarge understanding of these processes.

## Introduction

Chronic thrombo-embolic pulmonary hypertension (CTEPH) develops in a some patients after acute pulmonary embolism (1, 2). In CTEPH, pulmonary vascular resistance, initially increases due to the obstructions in the larger pulmonary arteries but is further elevated by pulmonary microvascular remodeling (1–3). CTEPH is defined as a mean pulmonary artery pressure ≥25 mmHg at rest persisting for at least 6 weeks in patients with previous pulmonary artery embolism (4). In addition to the increase in pressure, flow is redistributed toward the unobstructed parts of the pulmonary vasculature, causing local alterations in shear stress. Altered shear stress combined with systemic risk factors contribute to pathological processes like endothelial dysfunction, inflammation, vasoconstriction, and impaired vasodilation (5). In addition, these processes promote structural remodeling of both the obstructed and unobstructed pulmonary vasculature (6, 7). This remodeling leads to increased pulmonary vascular resistance that in turn augments afterload of the right ventricle (RV), thereby resulting in RV dilation and RV hypertrophy.

The degree of RV structural remodeling and functional adaptability have been demonstrated to be important determinants of functional capacity and survival in patients with CTEPH (8–10). In addition to RV remodeling, CTEPH is a risk factor for development of arrhythmias, especially atrial fibrillation (AF), which adds to the morbidity and mortality of patients with CTEPH (11, 12).

Yet, mechanisms involved in pulmonary microvascular remodeling, processes underlying the RV structural and functional adaptability in CTEPH as well as determinants of the susceptibility to arrhythmias such as AF in the context of CTEPH remain incompletely understood. Gathering knowledge about these processes has proven notoriously difficult, at least in part because (large) animal models with critical clinical features of human CTEPH and subsequent RV remodeling have only been developed relatively recently. In this review we will discuss current knowledge on the processes underlying development and progression of CTEPH, and on how porcine, canine and ovine models can help enlarge understanding of these processes.

## Definition, Prevalence, Incidence, and Clinical Phenotype of CTEPH

Pulmonary embolism results in an acute increase in pulmonary vascular resistance and a decrease in pulmonary vascular compliance, both of which contribute to an increase in RV afterload. CTEPH is defined as a persistent pulmonary artery pressure (PAP) above 25 mmHg, with a pulmonary wedge pressure below 15 mmHg, at rest for at least 3 months, despite therapeutic anticoagulation in order to discriminate this condition from “subacute” PE (4, 13–16). It is increasingly recognized that patients that fail to meet the PH cut-off value of 25 mmHg, yet have complaints similar to CTEPH patients, may have a milder form of the disease, so-called chronic thrombo-embolic disease (CTED) (3, 8, 17, 18). Indeed, PAP above 19 mmHg at rest following embolism are already associated with increased mortality at long term (15, 19). Mismatched perfusion defects on a ventilation/perfusion scan and specific diagnostic signs for CTEPH seen by multidetector CT angiography, MR imaging or conventional pulmonary cineangiography, such as ring-like stenoses, webs/slits and chronic total occlusions are imaging hallmarks for CTEPH.

The prevalence of CTEPH is still largely unknown. CTEPH develops in about 3–4% of patients after acute pulmonary embolism and in up to 10% of patients with recurrent pulmonary embolism (3, 20–22). The reported annual incidence of acute pulmonary embolism ranges from 750 to 2,700 per million adults (23–25). According to these numbers, the expected incidence of CTEPH would be 22.5–108 per million adults, while the reported numbers of diagnosed patients with CTEPH are substantially smaller. Three countries assessed the CTEPH incidence through nationwide registries. In the United Kingdom the CTEPH incidence was 1.75 per million (26), in Spain it was 0.9 per million adults (12) and in Germany it was 5.7 per million adults (27). Between ~300 and 400 patients were newly diagnosed with CTEPH per year in France and Germany (28, 29).

The symptoms of CTEPH are similar to those of other forms of PH, being shortness of breath, fatigue, syncope, chest pain, palpitations, and reduced exercise capacity, which (together with unawareness in many physicians' daily practice) contribute to the late diagnosis in a large number of patients. This delayed diagnosis negatively impacts the prognosis (30). Treatment options for CTEPH are limited and, even when treated, the disease often advances to right heart failure and even death. Proximal obstructions can be removed with surgical interventions such as pulmonary endarterectomy or balloon angioplasty (31–33) although these can only be performed in eligible patients. Therapeutic agents to modulate the pulmonary vascular resistance are limited to date. Riociguat, which is a soluble guanylyl cyclase stimulator, that activates the nitric oxide (NO) pathway without endogenous NO, thus acting as a vasodilator, inhibiting pulmonary smooth muscle cell growth and antagonizes platelet inhibition (i.e., preventing clot formation) is the only approved therapeutic agent in CTEPH to date (15, 31, 34–36).

## Large Animal Models of CTEPH

Over the past decades, the pathophysiology of CTEPH has been studied in large animal models. Different embolization protocols and materials including air, autologous blood clots, Sephadex beads, and glue have been utilized in swine, sheep and dogs (Table 1). Measurements in these models include measurement of PAP, with either indwelling (chronic), or Swan Ganz catheters. Cardiac output and stroke volume was measured using either the Swan Ganz catheter, a chronically implanted flow probe or *via* echo, CMR or PV loop catheter (Tables 1, 2). The PV loop catheter also allows measurement of right ventricular- pulmonary arterial (RV-PA) coupling, a measure of how well the right ventricle can cope with the increased afterload. In the absence of a PV-loop catheter, RV-PA coupling can also be assessed using the single beat method (59).

**Table 1:** Overview of large animal studies utilizing embolization techniques to create CTEPH/CTED, adapted from (37).

**Reference**	**Species**	**Sex**	**Embolic material**	**Embolizations (*N*)**	***N***	**PAP & CO assessment**	**Anesthesia during RHC**	**Recovery period**	**PAP (mmHg)**	**PVR (WU)**	**RVW/LVW + SW**	**V/Q scan/angiography**
Shelub et al. (38)	Canine	Female	Sephadex G50	Variable (16–30 weeks)	5	Catheter *via* jugular; dye dilution	None	>7 days	29 ± 4	8.3 ± 2.3	0.54^e^	NR
Perkett et al. (39)	Sheep	NR	Air (continuous)	12 days	5	Indwelling catheters + SG	None	1.5 h	23^f^ ± 2	5.2^f^	0.38 ± 0.06	Perfusion defects
Moser et al. (40)	Canine	NR	3–4 venous thrombi + Tranexemic acid	2	10	SG	Halothane	32 days	20.3 ± 2	4.2^a^	NR	NR
Weimann et al. (41)	Swine	Male	Sephadex G50 (15 mg/kg)	3	8	SG	Ketamine	7 days	18 ± 3	4.3^a^^,^^b^	NR	NR
Kim et al. (42)	Canine	NR	Ceramic beads (3 mm)	4	5	SG	Halothane	6 months	17 ± 2	4.3^a^	NR	Perfusion defects
Zhou et al. (43)	Sheep	Female	Air (continuous)	8 weeks	4	SG	None	7 days	34 ± 2.6	4.5 ± 0.9	0.36 ± 0.01	NR
Sage et al. (44)	Swine	NR	Right PA ligation	1	10	Open thorax cath & Transonic CO	Pento barbital	5 weeks	16.2 ± 1.3	10.05^c^ ± 0.69	NR	NR
Pohlmann et al. (45)	Sheep	NR	Sephadex G50 (~21.1 ± 0.5 g)	60	9	SG	none	1 day	35 ± 3	1.7 ± 0.2	0.42 ± 0.01	NR
Garcia-Alvarez et al. (46)	Swine	Male	Sephadex G50	4 (3–6)	9	SG	Midazolam	2 months	27 ± 3	2.2^d^ ± 1.1	NR	NR
Mercier et al. (47)	Swine	NR	Histoacryl + Left PA ligation	5	5	SG	NR	7 days	28.5 ± 1.7	9.8^a^	NR	Thrombi
Guihaire et al. (48)	Swine	NR	Histoacryl + Left PA ligation	5	5	SG + PV-loop	Isoflurane	6 weeks	41 ± 4	10.0^a^^,^^c^	NR	NR
Guihaire et al. (49)	Swine	NR	Histoacryl + Left PA ligation	5	13	SG + PV-loop	Isoflurane	7 days	34 ± 9	12.4^a^^,^^c^	NR	NR
Boulate et al. (50)	Swine	Male	Histoacryl + Left PA ligation	5	5	SG	NR	7 weeks	27 ± 1.1	7.9 ± 0.6	NR	NR
Aguero et al. (51)	Swine	Female	Sephadex G50 (20 mg/kg)	6	6	SG	Propofol	14 days	16 ± 2	1.5^b^	0.41 ± 0.02	NR
	Swine	Female	Sephadex G50 (20 mg/kg) + coiling	4	6		Propofol	1 month	23 ± 4	1.6^b^	0.47 ± 0.06	NR
Noly et al. (52)	Swine	NR	Histoacryl + Left PA ligation	5	5	SG + PV-loop	Propofol	14 weeks	26.8 ± 1.4	6.9 ± 0.6^a^^,^^c^	0.42 ± 0.05	NR
Tang et al. (53)	Canine	NR	Autologous thrombi (0.3^*^1 cm)	NR	13	Cath in PA	Propofol	14 days	25.2 ± 3.6	NR	NR	Perfusion defects
Rothman et al. (54)	Swine	Female	Ceramic beads (0.6–0.9 mm)	21–40	3	SG	Isoflurane	NR	36.6^g^ ± 0.9	NR	NR	NR
Rothman et al. (54)	Canine	Female	Ceramic beads (0.6–0.9 mm)	9–12	3	SG	Isoflurane	20 months^h^	47^g^	7.8	NR	Perfusion defects
Stam et al. (55, 56)	Swine	Either sex	LNAME + Microspheres 610–700 μm ~9000 per procedure	4 (2–5)	6	Indwelling cath	none	4–5 weeks	39.5 ± 5.1	7.8 ± 3.4	0.51 ± 0.03	NR
Mulchrone et al. (57)	Canine	Male	Sephadex G50 (~51250 ± 8189 spheres)	Every 3–4 days (4–8 months)	4	Indwelling cath	Propofol	14–84 days	34.3 ± 6.0	27.6 ± 5.0	NR	Perfusion defects
Loisel et al. (58)	Swine	NR	Histoacryl + Left PA ligation	5	6	SG + PV-loop	Propofol	6 weeks	26[23-28	6.6 [5.5–7.2]^a^^,^^c^	NR	NR

a Calculated from dynes·sec^−1^·cm^−5^;

b Calculated from indexed PVRi;

c Total pulmonary vascular resistance;

d Median (interquartile range) reported;

e Only reported 2/5 cases;

f Calculated from cmH_2_O or cmH_2_O·L^−1^·min;

g systolic PAP;

h *only reported of one animal. CMR, cardiovascular magnetic resonance; CPET, cardiopulmonary exercise testing; CT, computed tomography; LVW, left ventricular weight; NR, not reported; PA, pulmonary artery; PAP, mean pulmonary artery pressure; PV loop, pressure-volume loop; PVR, pulmonary vascular resistance; RHC, right heart catheterization; RVW, right ventricular weight; SG, Swan-Ganz catheter; SW, septum weight; WU, Wood units*.

**Table 2:** Overview of RV-functional measurements in CTEPH/CTED models.

**Reference**	**RV function assessment**	**Group**	**EDA or EDV**	**ESA or ESV**	**FAC or EF (%)**	**TAPSE (mm)**	**Ees/Ea**	**Fulton Index**
Guihaire et al. (49)	Echo, PV-loop	Sham	4.6 ± 0.6 cm^2^/m^2a^	NR	51 ± 8^a^	20 ± 2^a^	1.24 ± 0.17^c^	NR
		CTEPH	11.0 ± 2.4 cm^2^/m^2a^	NR	25 ± 4^a^	14 ± 4^a^	0.66 ± 0.18^c^	NR
Aguero et al. (51)	3D-Echo	Sham	101.7 ± 4.5 ml^a^	30.5 ± 2.9 ml^a^	70.0 ± 2.7^a^	24.5 ± 2.4^a^	NR	0.40 ± 0.03
		Embolisation	76.2 ± 14.1 ml^a^	23.3 ± 4.1 ml^a^	68.8 ± 7.0^a^	21.6 ± 2.9^a^	NR	0.41 ± 0.02
		Embolisaiton + coil	104.4 ± 21.0 ml^a^	43.3 ± 10.7 ml^a^	58.8 ± 3.7^a^	18.8 ± 2.4^a^	NR	0.47 ± 0.06
Noly et al. (52)	Echo, PV-loop	Sham	5.2 ± 0.2 cm^2^/m^2a^	NR	46 ± 2^a^	NR	1.39 ± 0.27^c^	NR
		CTEPH	9.7 ± 0.6 cm^2^/m^2a^	NR	26 ± 1^a^	NR	0.71 ± 0.15^c^	
Stam et al. (37)	Echo	Sham	554 ± 92 mm^2a^	207 ± 54 mm^2a^	54 ± 3^a^	24 ± 1^a^	NR	NR
		CTEPH	714 ± 83 mm^2a^	304 ± 69 mm^2a^	43 ± 4^a^	20 ± 2^a^	NR	NR
Stam et al. (55)	CMR	Sham	1.89 ± 0.25 ml/kg^b^	0.78 ± 0.12 ml/kg^b^	59 ± 2^b^	NR	2.35 ± 0.23^c^	0.40 ± 0.03
		CTEPH	2.31 ± 0.31 ml/kg^b^	1.24 ± 0.27 ml/kg^b^	48 ± 5^b^	NR	1.80 ± 0.15^c^	0.51 ± 0.03
Mulchrone et al. (57)	Echo, CMR	Sham	2.73 ± 0.06 ml/kg^b^	1.41 ± 0.07 ml/kg^b^	48 ± 5^a^	15 ± 1^a^	NR	NR
		CTEPH	2.98 ± 0.02 ml/kg^b^	2.05 ± 0.06 ml/kg^b^	32 ± 5^a^	8.5 ± 1^a^	NR	NR
Loisel et al. (58)	Echo, PV-loop	Sham	NR	NR	43.5 [33.8–45.4]^a^	21 [19.5–21]^a^	1.03 [0.92–1.05]^c^	NR
		CTEPH	NR	NR	32.8 [29.5–36.5]^a^	15.5 [13.8–17.3]^a^	0.69 [0.56–0.83]^c^	NR

a measured by echo;

b measured by CMR;

c *measured by PV-loop; Ees/Ea, indexof RV-PA coupling; FAC, fractional area change; EF, ejection fraction; TAPSE, tricuspid annular plane systolic excursion (mm); NR, not reported*.

Very few investigators succeeded in establishing CTEPH during prolonged follow-up although the PAP increases acutely upon embolization (51). Ideally, autologous thrombi should be to induce CTEPH, in order to mimic the contribution of potential factors released from and cells interacting with these thrombi. However, studies with autologous thrombi to induce CTEPH have generally failed, which has initially been ascribed to a more active fibrinolytic system in animals as compared to humans (3, 60). Plasminogen activator inbibitor-1 was upregulated in the endothelial cells lining the thrombus in humans (60). Furthermore, a prothrombotic milieu, evidenced by elevated levels of von Willebrand Factor and C-reactive protein is associated with CTEPH in humans (60) and CTEPH severity and prognosis correlate with circulating D-dimer levels (61). These conditions are generally difficult to mimic in animal models. Nevertheless, inhibition of the fibrinolytic system in animals with tranexamic acid did not result in successful establishment of CTEPH in dogs (40). This failure to maintain high PAP during follow-up suggests that many models resemble CTED (i.e., mean PAP between 20 and 25 mmHg) more than CTEPH (mean PAP ≥25 mmHg).

It should be noted however, that PAP is mostly assessed under general anesthesia, which due to its cardiodepressive effects, may have resulted in an underestimation of PAP. Prerequisites for successful induction of CTEPH appear to include repeated embolization procedures (37, 38, 43, 45–50, 54, 56, 57) and obstruction of at least 40–60% of the pulmonary vasculature (62, 63). In these studies, pulmonary artery pressure and pulmonary vascular resistance decreased in between embolization procedures, but showed a slow but progressive increase over time and remained elevated after cessation of the embolisations. Obstruction of a large part of the pulmonary vasculature has been performed in swine, by combined ligation of the left pulmonary artery and progressive embolization of the segmental arteries of the right lower lobe, to mimic the proximal obstructions induced by thrombo-emboli (47–50, 64). An alternative method is to use smaller (micro)spheres to obstruct the pulmonary vasculature. The relative amount of obstruction of the pulmonary vasculature can be estimated by comparing the amount of microspheres infused with the number of vascular branches of this size present in the pulmonary vascular bed. The latter can be estimated from the morphometrical description of the pulmonary vasculature, which, in humans contains 15 Strahler orders, from the capillaries to the main pulmonary artery (65). It has been proposed that the pulmonary vascular bed of dogs (10 kg) and rats (500 g) are 1 and 3 log orders smaller than those of humans (66). Extrapolation of these numbers to swine of 20 kg, the size most commonly used in embolization experiments, would result in a pulmonary vasculature that is ~0.5 log order, or 3-fold, smaller than that of humans. The porcine pulmonary vasculature showed 10 branching orders of vessels larger than 160 μm in swine (67). The number of 3rd order pulmonary small arteries (diameter 430 μm, range 380–570 μ) was estimated to be 2,100, while 590 4th order vessels (diameter 760 μm, range 660–990 μm) were estimated to be present (67). These numbers exclude supernumerary vessels, which are 1.6- (68) to 2.8-fold (69) more than conventional arteries. Taking into account these supernumerary vessels, a total of 5,460–7,980 arteries of order 3, and 1,530–2,240 arteries of order 4 are present in the porcine lung.

In the porcine studies from our laboratory, CTEPH was induced with multiple (up to 5) embolizations using an average of 9,000 microspheres of 600–710 μm in diameter per embolization procedure, hence 45,000 microspheres in total (37, 55, 56). This number is much higher than the number of order 3 and 4 pulmonary small arteries present in the porcine lung and hence should be able to obstruct a sufficiently large part of the pulmonary vasculature. Despite this large number of emboli, sustained CTEPH only developed when embolizations were combined with chronic inhibition of eNOS, suggesting that endothelial dysfunction is required for CTEPH to develop in swine. These findings are in accordance with a study by Rothman and co-workers, who used ceramic beads of similar size but failed to induce sustained CTEPH in swine (54). Conversely, when a similar protocol was used in dogs, CTEPH was successfully induced in the absence of eNOS inhibition (54). Other studies in dogs also show that repeated embolization (Sephadex beads of 100–300 μm in diameter) does result in sustained CTEPH (38, 57), but this requires a large number of embolizations over a period of several months. A total of ~50,000 beads was injected over a period of several months (57), which is similar to the number of beads required to induced sustained CTEPH in our swine model. However, it should be noted that these smaller beads penetrate deeper into the pulmonary vasculature, and that the number of pulmonary small arteries increases with decreasing size. Thus, in the human lung 67,400 vessels of order 5 (diameter 150 ± 20 μm), 285,000 vessels of order 6 (diameter 220 ± 30μm) and 86,000 vessels of order 7 (diameter 350 ± 90μm) 86,000 vessels are present. Particularly when taking into account the 0.5 log-order fold difference between dogs and pigs, the relative number of beads required to induce sustained CTEPH in dogs seems to be smaller as compared to pigs.

## Pulmonary Microvasculopathy in CTEPH

CTEPH not only involves the obstructed pulmonary arteries, but causes progressive microvascular remodeling of the non-obstructed distal pulmonary small arteries, which is associated with a further increase in resistance and decrease in compliance (1, 70), and contributes to worsening of CTEPH. The exact time-course of microvascular remodeling is unknown. It is difficult to distinguish between an increase in resistance due to recurrent embolizations and an increase in resistance due to microvascular remodeling even in animal models. Nevertheless, pulmonary vascular resistance continued to increase after cessation of the embolization procedures in our swine model, which suggest persistent remodeling of the distal vasculature (37, 56). Even after cessation of the eNOS inhibitor, which may also have contributed to a higher pulmonary vascular resistance by increasing microvascular tone, the high pulmonary pressure and resistance persisted (37, 56). This microvasculopathy was reversible upon reperfusion of the obstructed left lung vasculature (50).

Increased microvascular wall thickness results in narrowing of the lumen and stiffening of the pulmonary microvasculature. The mechanisms behind the pulmonary microvascular remodeling remain incompletely understood. A shift in balance of angiogenic factors, with decreased VEGF and high levels of angiopoietin 1 and 2 have been reported (1). Furthermore, chronic inflammation, evidenced by increased C-Reactive protein (CRP), as well as interleukin (IL)-10, monocyte chemotactic protein-1 (MCP-1), macrophage inflammatory protein-1α (MIP-1α) and matrix metalloproteinase (MMP)-9 was present in patients with CTEPH (71–73), indicating that chronic inflammatory processes may play a role in the microvasculopathy. In accordance with this concept, studies in swine showed an elevation in IL-6 expression in the unobstructed territory (50), while no change in IL-6 was found in obstructed territories (50) and a trend toward an increase in mixed territories (56). However, neither mRNA expression of interferon-γ, TNF-α, nor expression of TGF-β were altered in the lungs of swine with CTEPH (56). It is however likely that a general systemic inflammatory phenotype in patients with CTEPH induces endothelial dysfunction, thereby predisposing to pulmonary microvasculopathy.

The structural changes in the pulmonary microvasculature are accompanied by functional changes. These functional changes can be assessed using isolated pulmonary small arteries in animal models of CTEPH. These thicker wall of the non-obstructed pulmonary small arteries results in exaggerated vasoconstriction to both KCl and the thromboxane analog U46619 (56). In addition, microvascular remodeling induced alterations in two major endothelial signaling pathways, i.e., endothelin (ET) and NO. NO sensitivity was increased in pulmonary small arteries from swine with CTEPH. This increase in NO sensitivity was evidenced by an enhanced vasodilator response to the NO-donor SNP. Furthermore, while the vasodilator response to bradykinin was reduced, there was a similar attenuation of bradykinin-induced vasodilation by eNOS-inhibition. The increased NO sensitivity was therefore likely accompanied by and/or a consequence of a decrease in NO production (56). Loss of NO is in accordance with data from CTEPH patients showing reduced circulating NO levels and increased levels of the endogenous eNOS-inhibitor asymmetric dimethyl arginine (ADMA) (74). In addition, the vasodilator response to PDE5 inhibition with Sildenafil was reduced in isolated pulmonary small arteries from swine with CTEPH as compared to healthy controls (56). This reduced PDE5 activity likely reduced the rate of cGMP breakdown and therefore may have contributed to the increased NO-sensitivity. Furthermore, a reduction in endogenous PDE5 activity may explain that PDE5 -inhibition is less effective for treatment of CTEPH and underlines the importance of intervening more upstream in the NO-pathway either by administering NO (75) or stimulating soluable guanylyl cyclase (sGC) by Riociguat, which has recently been approved for therapeutic use in CTEPH (35, 76–78).

NO not only impacts the vasculature directly, but also indirectly by inhibiting the production of the potent vasoconstrictor ET. ET can induce vasoconstriction and vascular remodeling and plasma levels of ET are higher in both animal models (42, 56) and patients with CTEPH (50) and correlate with clinical severity (79, 80). Although beneficial effects of ET receptor antagonism (ERA) in preventing vasculopathy were shown in dogs (42) conflicting results of ERA therapy have been reported in CTEPH patients with some studies showing positive effects (81–83), whereas, the randomized, double-blind, placebo-controlled BENEFIT study, which investigated dual ERA therapy with Bosentan, showed no statistically significant effects of Bosentan in CTEPH patients (84).

The lack of clinical effect of ERA in CTEPH may be due to a decreased sensitivity of the pulmonary microvasculature to ET (56). This decreased sensitivity to ET was accompanied by an increased ET_A_ but decreased ET_B_ contribution to ET-induced vasoconstriction. These data were consistent with an increased ET_A_/ET_B_ gene expression ratio in lung tissue (56) as well as with the increased ET_A_ receptor expression in human pulmonary endarterectomy tissues (85) and in the lungs of swine with CTEPH (47, 50). These data from large animal models and humans suggest that ET signaling is altered in the pulmonary microvasculature in CTEPH. The loss of ET_B_ mediated vasoactive effects in isolated pulmonary small arteries (56) suggests that specific ET_A_ blockade alone would be more therapeutically efficacious. Although the AMBER trial, which investigated the ET_A_ specific ERA Ambrisentan was terminated early (ClinicalTrials.gov NCT02021292, NCT02060721), there are ongoing studies to ET_A_ specific ERAs such as Macitentan (MERIT) which is an ERA with high specificity for ET_A_ (86).

ET can signal *via* two pathways, the first one being calcium-dependent and involving phospholipase C (PLC)-mediated activation of myosin-light chain kinase. The second pathway is calcium-independent and encompasses RhoA-Rho-kinase mediated inactivation of myosin phosphatase (87). Although the Rho-kinase inhibitor Fasudil is therapeutically effective in some patients with PAH (88), knowledge about this pathway in the pathogenesis of CTEPH is limited. ET-induced vasoconstriction was mediated in part through Rho-kinase in isolated pulmonary small arteries from healthy swine, whereas, Rho-kinase inhibition had no effect in pulmonary small arteries from swine with CTEPH (56), implying that ET-induced vasoconstriction in CTEPH is mediated through the PLC-pathway. These data imply that microvascular constriction and remodeling occur *via* different pathways in CTEPH than in PAH in which the contribution of Rho-kinase was found to be augmented (89). Hence, using Rho-kinase inhibitors to alleviate pulmonary vasoconstriction and remodeling may not be efficacious in CTEPH patients.

## Characterization of Cardiac Remodeling in CTEPH

### Cardiac Dimensions and Function

**Figure 1:**
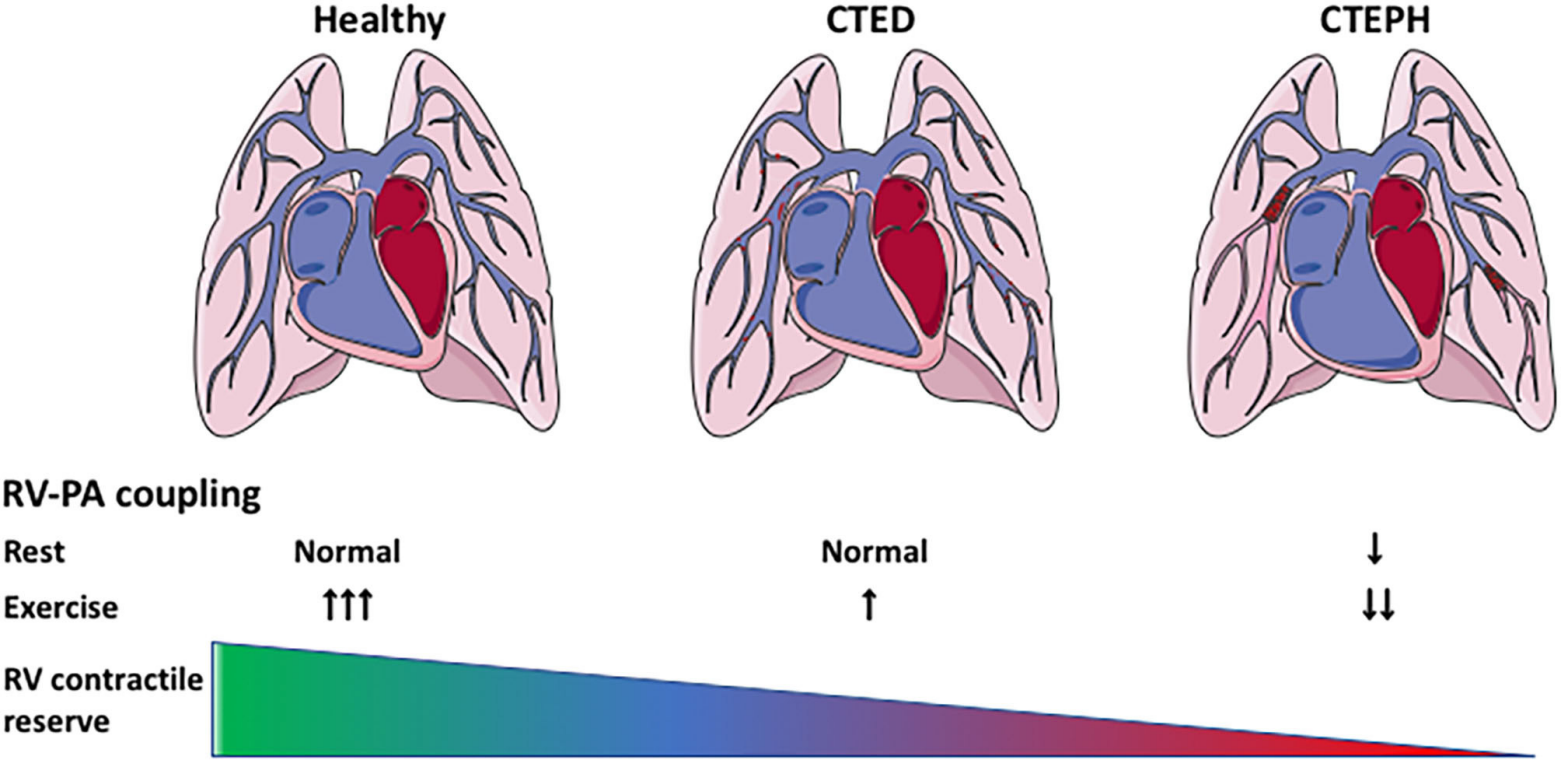
Cardiac remodeling and reserve in CTEPH. Cardiac remodeling in CTEPH, and the effects of exercise on the cardiac reserve. CTED, chronic thrombo-embolic disease; CTEPH, chronic thrombo-embolic pulmonary hypertension; RV-PA coupling: right ventricular-pulmonary artery coupling.

The afterload of the RV increases during development and progression of CTEPH. To cope with this increased afterload, the RV exhibits structural and functional changes to augment contractility (Figure 1). RV dilation and remodeling are dependent on the magnitude of the increase in afterload (51). The effects of CTEPH on cardiac structure and function have been examined using a variety of non-invasive, including ultrasound, CMR, CT, and invasive (pressure volume relation, assessment of RV-PA coupling) methods (for overview see Table 2). These studies show RV dilation (increase in enddiastolic area or volume) and a reduced contractile function, as evidenced by a decrease in fractional area change or ejection fraction, a decrease in TAPSE and a progessive decrease in RV-PA coupling in animals with CTED and CTEPH (Figure 1, for results of individual studies see Table 2).

RV remodeling is associated with cardiomyocyte hypertrophy, interstitial fibrosis and changes in capillary density (48, 55). RV hypertrophy, both in terms of RV weight and RV cardiomyocyte size was accompanied by activation of both pro- and antiapoptotic gene expression (upregulation of Caspase-3 and BCL2 mRNA, respectively). RV resting function is generally preserved, but BNP expression is increased (48, 49, 55), suggestive of an increased wall stress. The increased BNP showed a negative correlation with stroke volume and a positive correlation with global RV hypertrophy (48). RV structural and functional adaptability are important determinants of functional capacity and survival in patients with CTEPH (8–10).

Thus, reduced RV – PA coupling, which denotes the ratio of RV contractility and RV afterload, is associated with a lower exercise capacity (8) and patients with a dilated RV have a worse prognosis compared to patients in which RV function and geometry are preserved (90, 91).

It is increasingly recognized that in addition to RV systolic dysfunction, RV diastolic dysfunction is associated with a worse prognosis in patients with PAH (92). Diastolic dysfunction, evidenced by an increase in stiffness, is also present in pigs with type II pulmonary hypertension (93). Diastolic stiffness is determined by passive myocyte stiffness as well as interstitial fibrosis. Consistent with the findings in a rat model of pulmonary artery banding (94), the mild RV dysfunction in swine with CTEPH was characterized by an increase in the stiff titin isoform N2BA but not with changes in myocardial fibrosis as measured histologically. Furthermore, there was a change in the ratio between Col1 and Col3 in the RV, suggesting relatively more expression of the stiff Col1 isoform (55), which is consistent with data from rats with PAH (94), and may have contributed to a stiffer RV. Although, neither SERCA nor phospholamban gene expression were changed in our CTEPH swine model, it is possible that changes in their phosphorylation may play a role in altered Ca^2+^ handling, which is in turn implied to play a role in the development of RV dysfunction (93). Hence, future studies in relevant large animal models should investigate contractile function of individual cardiomyocytes as well as expression and phosphorylation of the contractile and calcium handling proteins SERCA, phospholamban, smooth muscle actin, titin, and troponins.

### Cardiac Inflammation, Oxidative Stress, Apoptosis, and Angiogenesis

A key factor that distinguishes compensated RV remodeling from RV failure is adequate myocardial perfusion (95). Angiogenesis is required to enhance RV perfusion commensurate with the increase in RV mass. Capillary density is preserved or even increased in adaptive RV remodeling, whereas, it is reduced in RV failure (95). Furthermore, RV maladaptive remodeling in CTEPH and PAH is accomanied by a reduction in myocardial perfusion reserve (96, 97). Conversely, capillary density was either unaltered (52) or increased in swine with CTEPH (55, 58), which is beneficial for myocardial perfusion and oxygenation, and suggests a state of adaptive RV remodeling. Nevertheless, HIF1α (52) and VEGFA-expression were higher in swine with CTEPH (52, 55, 58), suggesting that there was still a need for additional perfusion. Furthermore, VEGFA expression corelated inversely with RV-PA coupling (55) and further increasing capillary density using a therapeutic intervention with infusion of endothelial progenitor cells into the right coronary artery improved right ventricular function independent of changes in PVR (58). Hence, future studies should address myocardial perfusion and coronary flow reserve in different stages of RV (mal) adaptation and assess whether RV adaptations is different in CTED vs. CTEPH.

**Figure 2:**
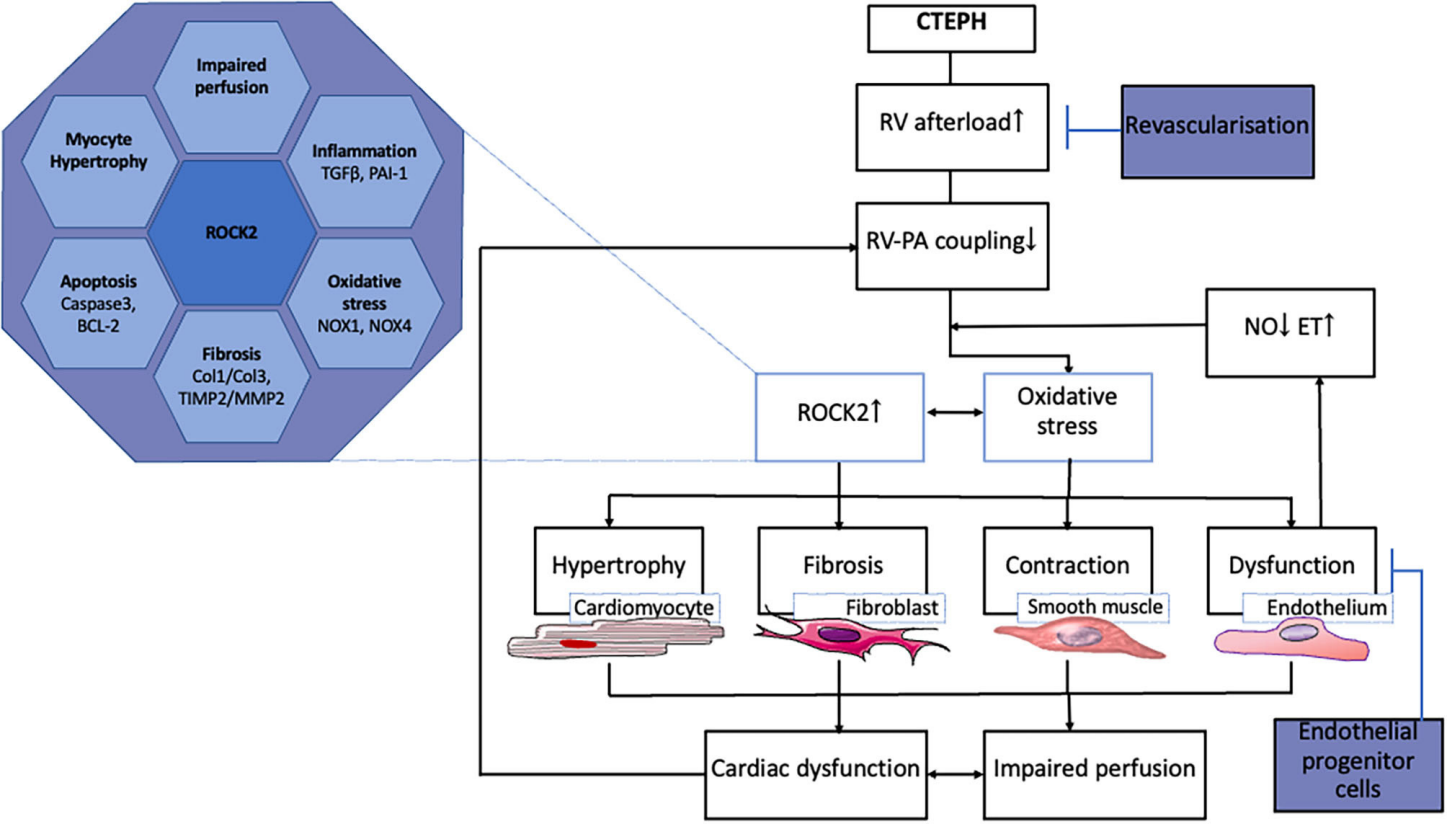
Processes involved in RV remodeling in CTEPH and therapeutic interventions tested in animal models.

The transition from compensated RV remodeling to RV failure is also associated with inflammation and activation of the immune system (98–100). Although, the mRNA of genes involved in immune modulation (TNF-α, IL-6, IFN-γ) was not altered in the RV of swine with CTEPH, expression of TGF-β was higher (52, 55). Activation of the TGF-β pathway is known to play a pivotal role in the development of PH (101, 102). TGF-β pathway activation was further supported by the higher expression PAI, a downstream target of TGF-β signaling in these animals. Just like endothelin, activation of the TGF-β pathway can result in activation of the Rho-kinase pathway (103–105). Indeed, ROCK2 expression was upregulated in the RV of swine with CTEPH. Importantly, ROCK2 activation is involved in cardiac hypertrophy, oxidative stress, angiogenesis, apoptosis, and fibrosis and may therefore present a major deleterious factor in RV-remodeling (106–108) (Figure 2). In addition, ROCK2 activation increases phosphorylation of protein phosphatase 1 (PP1), which in turn regulates Ca^2+^ handling and Ca^2+^ sensitivity in the cardiomyocytes (108).

ROCK2 is also expressed in the coronary vasculature, where it is associated with oxidative stress and NOX-expression (109). NOX1, NOX2, and NOX4 expression was elevated in the right coronary artery of swine with RV pressure overload due to pulmonary artery banding. The increased expression of these NOX- isoforms was accompanied by oxidative stress and endothelial dysfunction, notwithstanding unaltered eNOS expression (110). Furthermore, an increase in circulating NOX4 has been shown in patients with PAH (111). The upregulation of NOX1 and NOX4, and the unaltered eNOS expression in the RV of swine with CTEPH are consistent with these data (55). Interestingly, expression of NOX1 and NOX4 as well as ROCK2 correlated inversely with RV-PA coupling, suggesting that oxidative stress in the myocardium may contribute to deterioration of RV-function (55).

Hence, it remains to be investigated whether Rho-kinase inhibition may prevent adverse cardiac remodeling despite observations that it has no beneficial effects on pulmonary vascular tone and remodeling. Furthermore, animal models may be well-suited to further establish markers of RV dysfunction.

## Cardiac Arrhythmias in the Context of CTEPH

### Epidemiology and Clinical Implications

Although arrhythmias have been identified as relevant contributors for morbidity and mortality in patients with CTEPH, only little is known about the incidence and prevalence of arrhythmias in this patient cohort (112–115). Besides the very limited number of studies performed in general, these studies also included patients with other etiologies leading to pulmonary hypertension (in most cases PAH type 1) which makes it challenging to provide data specifically in patients with CTEPH. In general, supraventricular arrhythmias are more common in patients with CTEPH than in the general population ranging from 11.7 to 22% in retrospective studies (115, 116) and from 13.4 to 25.1% in prospective studies (11). The most common arrhythmias observed in these studies were atrial fibrillation (AF) and atrial flutter (AFlut). Kanemoto studied 101 patients with PAH and observed arrhythmias in 17.8% (113). The most frequent arrhythmias in this cohort were (i) sinus tachycardia (38%), (ii) sinus bradycardia (18%), and (iii) first degree AV block (15%) with sinus tachycardia being significantly associated with mortality. Ventricular arrhythmias such as ventricular tachycardia (VT) or ventricular fibrillation (VF) have been demonstrated only in about 8% of patients with PAH and sudden cardiac death (SCD) (117). Due to the pathophysiologic alterations in PAH and the observation of VF and VT in a rat model of PAH (118, 119) one would expect a higher incidence of VT/VF in PAH patients potentially indicating difficulties in diagnosing ventricular arrhythmias due to a lack of permanent ECG monitoring (e.g., implantable loop recorders) in such patients (12). Even more frequent than specific arrhythmias are ECG abnormalities in PAH patients. Tonelli and colleagues evaluated ECGs over time in 50 patients with PAH and could see a significantly increased heart rate as well as prolonged PR interval, QRS duration and QTc duration in terminally diseased patients (120) which was further confirmed by Rich et al. (121). They also observed that none of the patients had a “normal” ECG before death and demonstrated a correlation between QRS/QTc prolongation and impaired RV function, increased RV mass and poor prognosis with a QTC > 480 ms being an independent predictor for mortality (120). Occurrence of arrhythmias is associated with clinical decompensation, although, it is incompletely understood whether arrhythmias occur as a cause or consequence of clinical worsening or both. However, conversion to sinus rhythm improved clinical status and in most cases reverses cardiac decompensation, whereas, the presence of sustained supraventricular arrhythmias is associated with a worse prognosis (11, 12). Overall, supraventricular arrhythmias are associated with increased mortality in PAH patients (3.81-fold increased hazard of death) (122).

### Arrhythmia Mechanisms

The increased vulnerability to arrhythmias in CTEPH is incompletely understood. So far, our knowledge on CTEPH-associated arrhythmogenesis is based on clinical findings in patients. So far, no specific animal model for CTEPH has been investigated with respect to arrhythmias, only in the monocrotaline induced PAH models in rat and dog arrhythmogenesis has been studied. Thus, only extrapolated mechanistic data from non-CTEPH animal models or patients/animal models with non-CTEPH RV dysfunction or cardiac remodeling leading to the clinical phenotype seen in CTEPH patients are currently available and a specific mechanistic understanding of CTEPH-mediated electrophysiologic alterations and arrhythmogenesis is lacking.

Having this major limitation in mind several arrhythmia mechanisms have been suggested in CTEPH, including autonomic, electrical, or structural remodeling (12). Autonomic remodeling in PAH results in decreased heart rate variability, an overall increased sympathetic activity and an adrenergic remodeling in the RV including downregulation of β1-, α-, or dopaminergic receptors. This autonomic dysregulation is clinically reflected by arrhythmias (123) but also by sinus tachycardia which may be a reactive mechanism to maintain RV output (124). The influence of the autonomic system on arrhythmogenesis has been further demonstrated in a dog model of PAH (125). Eight weeks after dehydromonocrotaline treatment beagles develop PAH and show an increased vulnerability to AF/AFlut. They could confirm a downregulation of β1 receptors in RV, but in RA they found an upregulation and an increased density of sympathetic nerves. Finally, they ablated the ganglionated plexi which resulted in reduced AF/AFlut inducibility and further confirms the important role of the autonomic nervous system in PAH-associated arrhythmogenesis.

In patients with PAH increased RV stretch, hypertrophy and fibrosis have been demonstrated – well-known proarrhythmic mechanisms summarized under the term “structural remodeling.” These structural alterations result in reduced conduction velocity and the occurrence of re-entry (126). In addition to that, both in patients and a rat model for PAH electrical alterations (so called electrical remodeling), can be observed including downregulation of potassium channels, connexin-43, or changes in calcium handling leading to conduction slowing, QTc and action potential duration prolongation, or afterdepolarizations and ectopic activity (119–129). All these mechanisms finally result in an increased susceptibility of arrhythmias (126) but their relevance in CTEPH remains to be established.

## Cardiopulmonary Stress Testing

**Figure 3:**
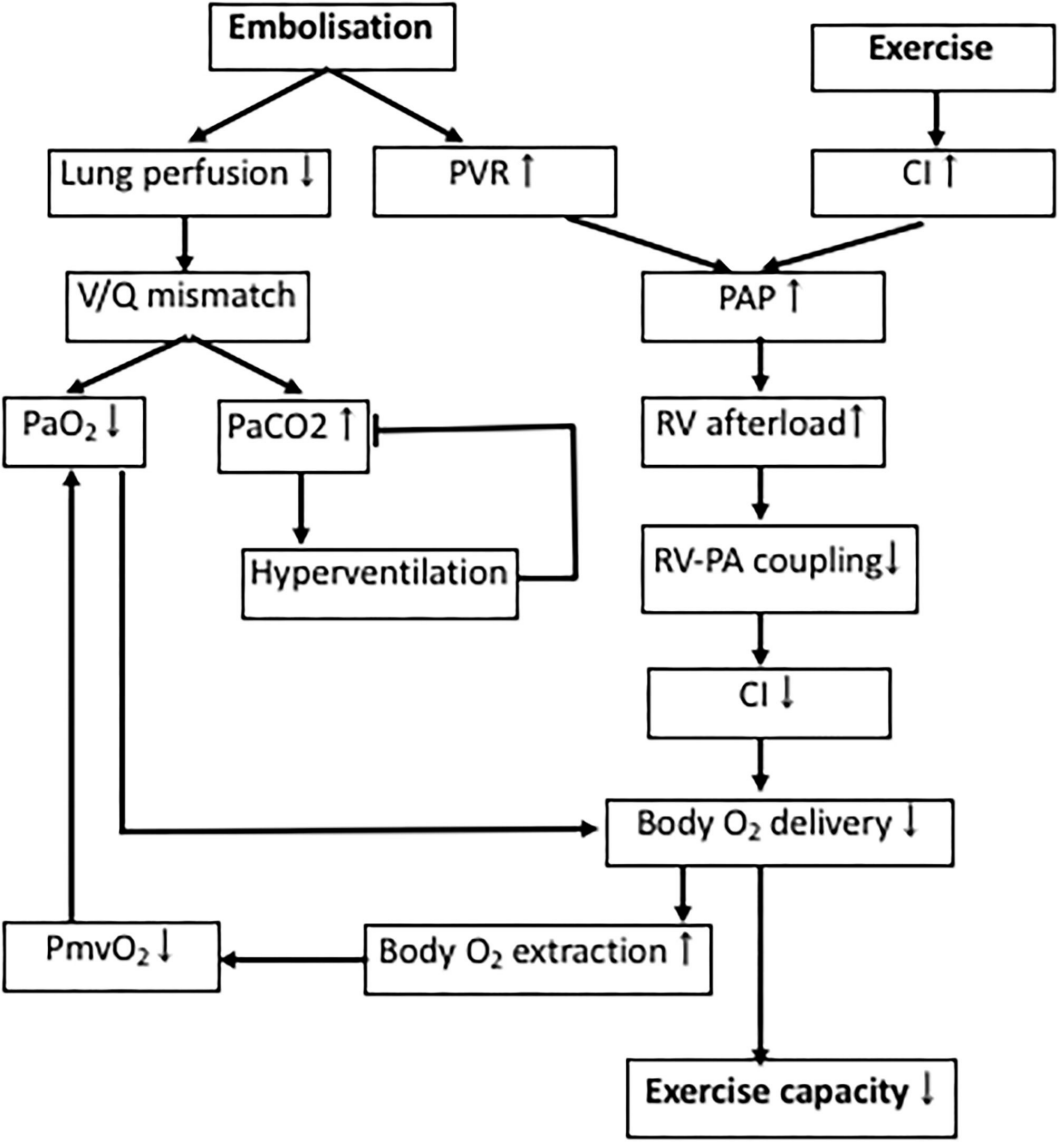
Determinants of exercise intolerance in CTEPH. Processes involved in decreased exercise tolerance in CTEPH. PAP, pulmonary artery pressure; PVR, pulmonary vascular resistance; RV, right ventricle; V/Q-mismatch, ventilation/perfusion-mismatch; P_mv_O_2_, mixed venous oxygen pressure; P_a_O_2_, arterial oxygen pressure; P_a_CO_2_, arterial carbon dioxide pressure.

Exercise testing after pulmonary embolism is predictive of development of PH and/or patient outcome in established CTEPH (130–133). Both patients with CTED and CTEPH show impaired exercise tolerance (8, 18, 134, 135). In accordance with these human data, swine with CTEPH also showed evidence of exercise intolerance (37, 55). This impaired exercise capacity is principally caused by an exacerbated increase in PAP and pulmonary vascular resistance during exercise, that further increases RV afterload and the V/Q-mismatch in the lungs (Figure 3) (90, 134, 135).

Physiological dead space is increased in proportion to the increase in PVR in CTEPH, resulting in a lower P_a_O_2_, which results in a reduction in P_a_CO_2_, due to compensatory hyperventilation (136). Similarly, the slightly lower P_a_O_2_ in swine with CTEPH is consistent with dead space ventilation, a mild V/Q mismatch, and a subsequent decrease in capillary transit time at rest. However, P_a_CO_2_ was not altered in the swine with CTEPH neither at rest, nor during exercise (37). This discrepancy in CO_2_ response may in part be due to the observation that swine lack collateral ventilation and therefore cannot equalize intraregional V/Q differences between alveoli. Furthermore, healthy quadrupeds already ventilate and perfuse their entire lungs at rest (137), and hence cannot further recruit hypoventilated lung areas and improve V/Q mismatch by increasing ventilation. Thus, hyperventilation may not be capable of reducing P_a_CO_2_ levels below normal as seen in humans (37, 56). The lower P_mv_O_2_ observed in CTEPH is partly a consequence of the lower P_a_O_2_ due to the V/Q mismatch, and in part reflects the decreased blood flow due to a lower cardiac index (CI), forcing the body to extract more oxygen.

In accordance with the studies in CTED and CTEPH patients in 2015 (8, 18), the RV of swine with CTEPH was not able to cope with this increased afterload evidenced by the limited exercise-induced increase in stroke volume and CI (Figure 1). Furthermore, RV-PA coupling was reduced in swine with CTEPH (49, 55, 64) and a correlation was found between reduced coupling and a reduced SV reserve with dobutamine in swine (49) as well as between afterload and RV-PA coupling (55). Importantly, recent studies in patients with CTEPH show that RV-PA coupling correlates with exercise capacity (8), which in turn is a strong predictor of clinical outcome (90).

During exercise at 4 km/h, the anaerobic threshold was reached in swine with CTEPH and maximal body oxygen consumption was reduced which is consistent with a reduced exercise tolerance in CTEPH patients (18, 111, 132, 138). This reduction in maximal oxygen consumption was principally due to a decreased CI (27% lower as compared to healthy) and to a lesser extent to a decreased arterial oxygen content (10% lower as compared to healthy). Hence, exercise intolerance in CTEPH is principally caused by the increased pulmonary vascular resistance, which augments afterload of the RV, and thereby limits the exercise induced increase in CI. The relative contributions of cardiac and pulmonary dysfunction to the exercise intolerance in patients with more severe RV dysfunction remains to be established.

## Added Value of Animal Models

CTEPH is associated with increased mortality and therefore a clinically highly relevant disease. The underlying pathophysiology is complex and currently poorly understood. Animal models are invaluable in this regard to study causal mechanisms and to identify potential targets for drug development or establishment of diagnostic/prognostic biomarkers.

In this context, CTEPH large animal models are of special interest since chronic instrumentation or repeated measurements are possible and they better resemble pathophysiologic hallmarks of the human situation such as microvascular remodeling, as well as remodeling and/or electrophysiologic alterations of the RV in CTED as well as CTEPH (37, 55, 56, 139–141).

Thus, those close-to-human large animal models (37, 55, 56, 139) allow to identify and to validate biomarkers (e.g., circulating biomarkers in the blood, hemodynamic biomarkers or ECG traits) to facilitate early detection of disease and/or disease progression in patients which could lead to the development of preventive strategies in the future as well. Also, in large animal models interventions that interfere with microvascular and/or RV remodeling and have direct clinical applicability (e.g., catheter-based approaches) are possible to get more insight in the pathways involved in this remodeling and get more specific targeted therapy for inoperable CTEPH patients or patients with residual PH after surgery. An example of such treatment to be tested for improvement of RV function based on swine data is ROCK2 inhibition. Thus far, few studies in large animal models have been performed to study the effect of therapeutic interventions. One study investigated lung revascularization (50) and one study investigated the effect of endothelial progenitor cells to improve RV perfusion (58) (see also above).

In addition, animal models may also be used to delineate sex-differences that are known to exist in development, progression and possibly interventions of CTEPH (142) and shed more light on the importance and implications of cardiopulmonary exercise testing in CTED and CTEPH patients. Finally, electrophysiologic evaluation beyond pure mechanistic concepts (which can be obtained in rodents or small animals) and especially studying novel anti-arrhythmic therapeutic approaches are more feasible in larger animals since the electrical properties of the heart are more similar to humans (compared to rodents) (140). Some innovative agents targeting pulmonary vasculature are indeed currently under clinical investigation (ClinicalTrials.gov NCT03689244, NCT01416636, NCT03809650, NCT03273257, NCT02634203, NCT00910429) but have not been tested in large animal models of CTEPH.

## Author Contributions

KS drafted the manuscript. SC, YT, and DM revised the manuscript. All authors approved the final version of the manuscript.

## Conflict of Interest

The authors declare that the research was conducted in the absence of any commercial or financial relationships that could be construed as a potential conflict of interest.
